# Electrode and dielectric layer interface device engineering study using furan flanked diketopyrrolopyrrole–dithienothiophene polymer based organic transistors

**DOI:** 10.1038/s41598-020-76962-x

**Published:** 2020-11-17

**Authors:** Basanagouda. B. Patil, Yasunori Takeda, Subhash Singh, Tony Wang, Amandeep Singh, Thu Trang Do, Samarendra P. Singh, Shizuo Tokito, Ajay K. Pandey, Prashant Sonar

**Affiliations:** 1 School of Electrical Engineering and Robotics, Science and Engineering Faculty, Queensland University of Technology (QUT), Brisbane, QLD 4000 Australia; 2 School of Chemistry, Physics and Mechanical Engineering, Science and Engineering Faculty, Queensland University of Technology (QUT), Brisbane, QLD 4000 Australia; 3 Research Center for Organic Electronics (ROEL), Yamagata University, 4-3-16 Jonan, Yonezawa, Yamagata 992- 8510 Japan; 4 Department of Physics, School of Natural Sciences, Shiv Nadar University (SNU), Gautam Buddha Nagar, Uttar Pradesh 201307 India; 5 Centre for Material Science, Queensland University of Technology, Brisbane, QLD 4000 Australia

**Keywords:** Electronic and spintronic devices, Materials for devices

## Abstract

We successfully demonstrated a detailed and systematic enhancement of organic field effect transistors (OFETs) performance using dithienothiophene (DTT) and furan-flanked diketopyrrolopyrrole based donor–acceptor conjugated polymer semiconductor namely PDPPF-DTT as an active semiconductor. The self-assembled monolayers (SAMs) treatments at interface junctions of the semiconductor–dielectric and at the semiconductor–metal electrodes has been implemented using bottom gate bottom contact device geometry. Due to SAM treatment at the interface using tailored approach, the significant reduction of threshold voltage (V_th_) from − 15.42 to + 5.74 V has been observed. In addition to tuning effect of V_th_, simultaneously charge carrier mobility (µ_FET_) has been also enhanced the from 9.94 × 10^−4^ cm^2^/Vs to 0.18 cm^2^/Vs. In order to calculate the trap density in each OFET device, the hysteresis in transfer characteristics has been studied in detail for bare and SAM treated devices. Higher trap density in Penta-fluoro-benzene-thiol (PFBT) treated OFET devices enhances the gate field, which in turn controls the charge carrier density in the channel, and hence gives lower V_th_ = + 5.74 V. Also, PFBT treatment enhances the trapped interface electrons, which helps to enhance the mobility in this OFET architecture. The overall effect has led to possibility of reduction in the V_th_ with simultaneous enhancements of µ_FET_ in OFETs, following systematic device engineering methodology.

## Introduction

A very low cost, low powered and flexible integrated circuits are some of the important basic requirement for the future advanced technologies including internet of things (IoT), robotics, wearable electronics, and fourth industrial revolution. In order to realize the real world applications such as actuators and sensors in robots^1,2^, wearable bio electronics and healthcare^3^, the overall performance of OFETs should be higher. With future applicability in such high-end applications, and for OFETs overall effective diffusion, there has been an increasing necessity of understanding the possible reasons for the enhancement of mobility values and controlled reduction of V_th_ simultaneously, upon following device engineering methodology. Once the low powered OFET devices, with enhanced speed of operation are achieved, the reasons for such a change in phenomenon needs to be analysed for better standardizations. This can be more effectively possible, when a similar class of co-polymer is chosen, and the behavioural change is re-proven with systematic study. Additionally, the physics behind of phenomenon for such change must be understood for more clarity and utilization. Accordingly, in our previous work we have shown the methodology, which showcases that effective SAM treatment at interfaces can lead to enhanced mobility, and reduced threshold voltage^4^. In this work, we highlight that achievement of similar behaviour upon surface treatment is repeatable with a different copolymer. Along with that, the reasoning for the such a change in device behaviours, due to varied extent of trap states upon surface treatment in discussed.

The essential surge, in the performance of the OFETs can be modulated via thin film interface engineering using chosen organic semiconductor material^5^. Accordingly, there are various ways to enhance the overall OFETs performance, including the development of new organic donor–acceptor semiconductors^6–10^, relevant semiconductor doping^11^, implant doping^12^, use of high dielectric constant materials^13,14^, various material deposition processes^15,16^, and use of self-assembled monolayers (SAM) treatments^4,5,17,18^. In most of the above-mentioned strategies, it has been highlighted that charge carrier transport in polymer thin films is largely influenced by the structural and energetic disorders. These are generally introduced into the films and/or device interfaces^19^. Mainly, the interaction between the dielectric layer and the semiconductor thin films has a direct impact on the orientation of the molecular segments in the channel region of the OFETs and thereby such interaction should be feasible to support efficient charge transport across channel^13^. Accordingly, the interaction between dielectric and semiconductor layer is widely addressed through the appropriate choices of the SAM material, and its effective treatment on the dielectric surface before the semiconductor material is deposited on top of dielectric surface^20^. Among many possible silanes based SAM treatments on the SiO_2_ surface, octyl trichlorosilane (OTS) is one of the most widely used^21–23^ due to its appropriate alkyl chain length which enhances alkyl–alkyl interdigitation between the SAM alkyl chain and alkyl chain substituted on the conjugated backbone motifs.

The interface engineering to optimize the charge injecting contacts becomes important to realize high performing OFETs. SAM treatments like PFBT on the surface of the source and drain metal electrodes have an impact on the charge injection possibilities, because of their polar nature. PFBT tend to shift the position of the work-function based on the terminating group present in its backbone^4^. This will make the charge injection from the respective electrode adjustable based on the band gap of the respective organic semiconductor material^24^. Adjusting the work function of an electrode is critical to modulate the charge transport and electrical performance because the threshold voltage (V_th_) shifts in OFETs is influenced by the intrinsic electric field produced by the permanent dipole of the SAM layers and by the electrochemical reaction between surface functional groups and the semiconductor molecules^25^. Basically, both such modifications can variably influence the crystallization process of the active layer, in terms of molecular packing and grain boundary sizes independently. Thereby, the study of their combined effect with proper reasoning about the underlying physics, on some of those high performing organic semiconductor based OFETs has become essential^24–27^.

In our previous report, we have demonstrated that by introducing long-branched alkyl groups to the donor–acceptor (D–A) polymers, their solution processability can be improved and the molecular packing in the film state can be reduced^21^. This design, which has been intensively studied, leads to a significant enhancement of hole mobility approaching level of (12 cm^2^ V^−1^ s^−1^)^28^and even exceeding (20 cm^2^ V^−1^ s^−1^)^29^. Among reported various donor–acceptor (D–A) based copolymers, recently diketopyrrolopyrrole (DPP) dye incorporated molecular and polymeric D–A materials have illustrated outstanding performance in transistor based various organic electronic devices including stretchable transistors, ambipolar transistors, phototransistors, chemical sensors and so on^30^. The lower cost, easy synthesis, solution processability, ink forming capability ample scope for chemical structural modification, enhancing π–π stacking due to aromatic fused lactam structure makes DPP building block as a star conjugated moiety. The energy levels consisting of both the highest occupied molecular orbital (HOMO) and lowest unoccupied molecular orbital (LUMO) and its subsequent band gap can be systematically tuned either by varying flanking groups attached to the DPP core or by incorporating comonomers (which are either strong/weak electron donor or acceptor) in the main conjugated backbones. Either holes or electrons can be injected into their respective HOMO or LUMO determines their nature either as p-type or n-type materials for transistor. If the both holes and electrons are getting injected simultaneously into their HOMO and LUMO’s due to lower band gap nature, then they can be potential materials for ambipolar transistors since their energy levels are close to the work function of metal electrode. There are numerous numbers of flanking groups such as furan^31^, thiophene^32^, selenophene^33^, pyridine^34,35^ thiazole^36^, thienothiophene^35^ and naphthalene have been used in the literature for the polymer synthesis. Among the reported DPP molecular and polymer semiconductors, thiophene flanked DPP is one of the most widely studied building block^37^. Taking benefits of biodegradability of furan and its higher solubility upon combining with large extended conjugated comonomer blocks, recently, furan flanked DPP (DPPF) materials have attracted great deal of attention for designing solution processable organic semiconductor materials with high performances^38,39^.

Compared to DPPT^39,40^, DPPF has smaller oxygen heteroatom present in the furan ring and shorter due to this, the C = O bond may lead to less steric hindrance between the DPP unit and the neighbouring aryl group and might allow more co-planar structure^41^. Herein, a D–A polymer based on DPPF as acceptor and DTT as a donor was synthesized and investigated as active channel semiconductor for OFETs devices.

In our current work, we study the effects altered trap density upon the surface modifications on the PDPPF-DTT based OFETs. To understand the reasoning of enhancement in mobility after OTS treatment, we summarize its correlation with the observation from the thin film Atomic Force Microscopy (AFM), and thin film X-Ray Diffraction (XRD) analyses. The correlation of enhancement of mobility and reduction of V_th_ upon these surface treatments is explained through the energy level band diagram. XRD analysis confirms that, after OTS treatment, the morphology of the semiconducting layer improved which help to increase the mobility. Then we showcase that the interface traps mainly affect the gate field, which in turn control V_th_ of the OFET device. This effect leads to the mobility governance by both the fields, and the morphology control by SAM treatment, on this type of polymers based OFETs.

## Results and discussion

**Figure 1:**
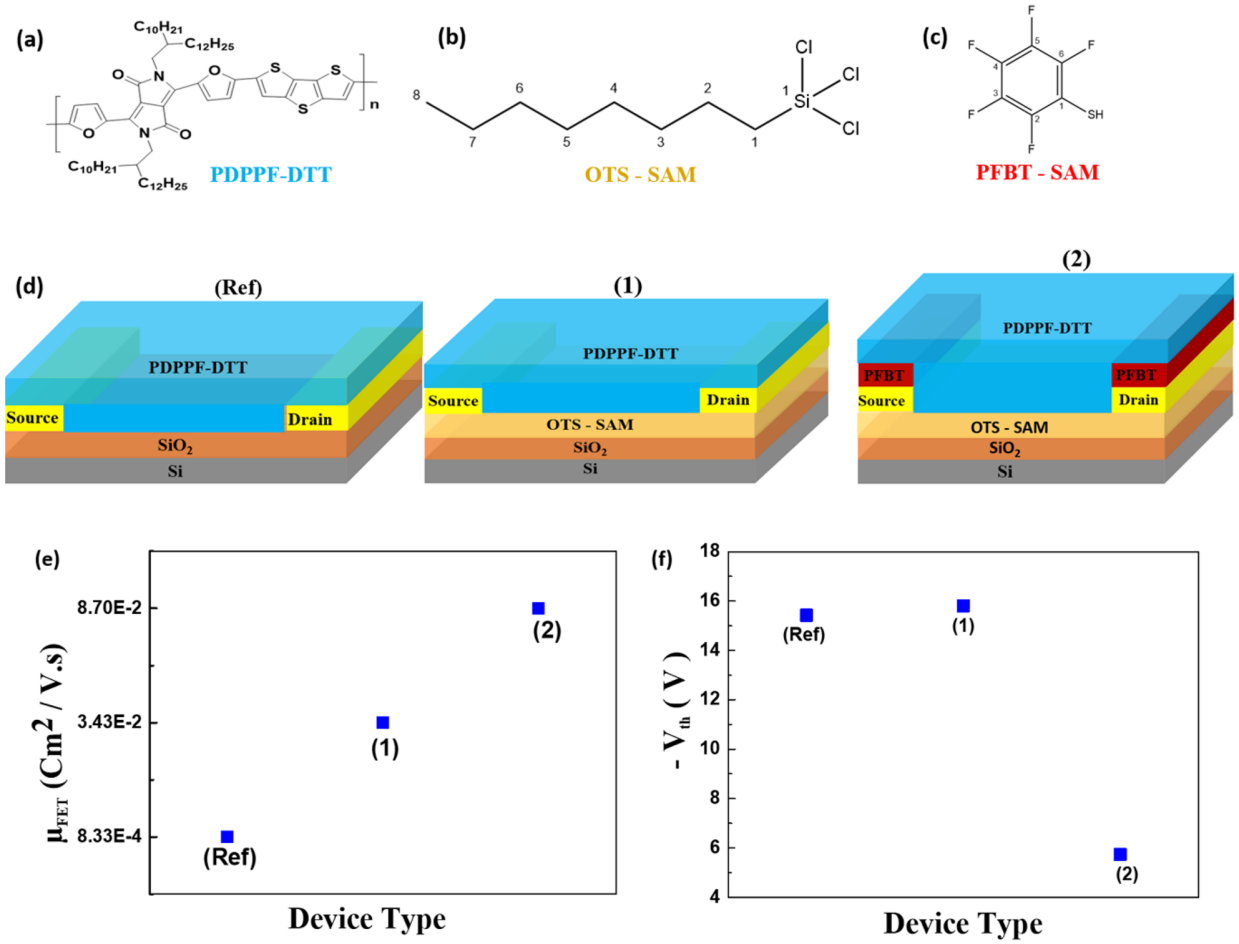
The chemical structures of (**a**) PDPPF-DTT, (**b**) OTS, (**c**) PFBT, and (**d**) the device structures of OFETs analysed. (Ref) is a considered reference device, wherein, there is not any kind of SAM treatment applied to SiO_2_ and Au source–drain (S/D) contacts. Device mentioned as (1) is with OTS treated SiO_2_, and device mentioned (2) is the one with both PFBT and OTS treatment at Au and SiO_2_ surface respectively. Finally (**e**) and (**f**) describe average µ_FET_ and V_th_ on different device types respectively.

Figure 1a shows molecular structures PDPPF-DTT polymer whereas Fig. 1b,c shows the molecular structures of OTS and PFBT SAMs materials used for SiO_2_ and S/D contact treatment, respectively. The systematic devices structures are planned based on the lines of those discussed in our previous work, using PDPPT-DTT^4^ polymer. In this study, we utilise the PDPPF-DTT version of the polymer. The OFET device structure are categorized into three types. Firstly, as in Fig. 1d, we fixed the bare (untreated) SiO_2_ and untreated S/D OFETs as the reference (Ref.) device type. Secondly, we consider Fig. 1d. (1) as the OFETs device type whose SiO_2_ was treated with OTS leading to a hydrophobic surface with a contact angle of 106º, on this device type there is not any PFBT treatment done. Lastly, we consider Fig. 1d. (2) as the OFETs device type wherein, along with the OTS treatment on SiO_2_, there is additional PFBT treatment of gold (Au) S/D electrode.

Figure S1(a), Fig. S1(b), and Fig. S1(c) shows the 3-dimensional chemical structures of PDPPF-DTT polymer, along with that of OTS, PFBT respectively. Figure 1e,f describes the variation in mobility and threshold voltages in all three device architectures. Which clearly depicts that upon OTS and PFBT treatment, PDPPF-DTT polymer based OFET device performance is better both in terms of µ_FET_ enhancement and lowered V_th_.

**Figure 2:**
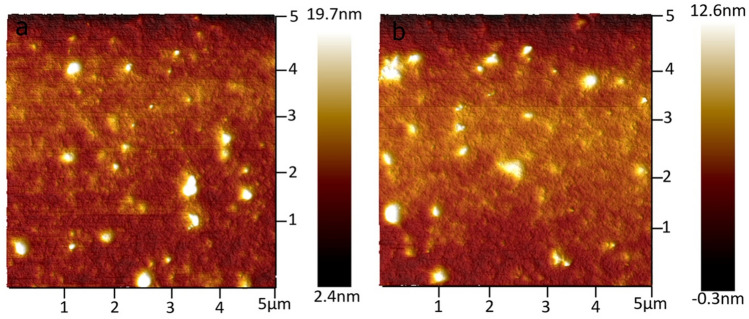
AFM images (5 µm × 5 µm surface area, and scale is + 10 nm to − 10 nm) of PDPPF-DTT, after annealing at 150 °C for 30 min with average nominal height of (**a**) 3.68 nm for untreated, and (**b**) 4.42 nm for OTS treated SiO_2_ surfaces, respectively.

We characterize the bare (untreated) SiO_2_ and OTS treated SiO_2_ surfaces in terms of hydrophilicity and morphology of the deposited active semiconductor thin film. Fig. S8(a) and Fig. S8(b) shows the contact angle measurement for bare and OTS treated SiO_2_ surface, respectively. The contact angle, as discussed increases from 28° to 106° after OTS treatment, this clearly indicates the formation of hydrophobic surface after OTS treatment. This aids in the process of reduction of hysteresis in transfer characteristics and lowered trap density in OTS treated surfaces and discussed in the further section below. AFM images of PDPPF-DTT thin film grown over bare surface is illustrated in Fig. 2a, Fig. S6, and Fig. S8(c). The one on OTS treated SiO_2_ surface is illustrated in Fig. 2b, Fig. S7, and Fig. S8(d). The film is annealed at 150 °C for 30 min in both the cases directly on the top of the hot plate in an N_2_ environment. Images captured in tapping mode by scanning the 5 µm × 5 µm surface area. The RMS average nominal height of the film grown over bare SiO_2_ surface is 3.68 nm and is 4.42 nm for the film grown over OTS treated surfaces. Such an mild increased film height on average, in conjunction with increased contact angel on OTS treated SiO_2_ provides a better interface between the polymer and the dielectric interface^42^, promoting the molecular ordering of the overlaid channel semiconductor^43^. Thus, leading to enhanced field-effect mobilities after OTS treatment in PDPPF-DTT based OFETs.

**Figure 3:**
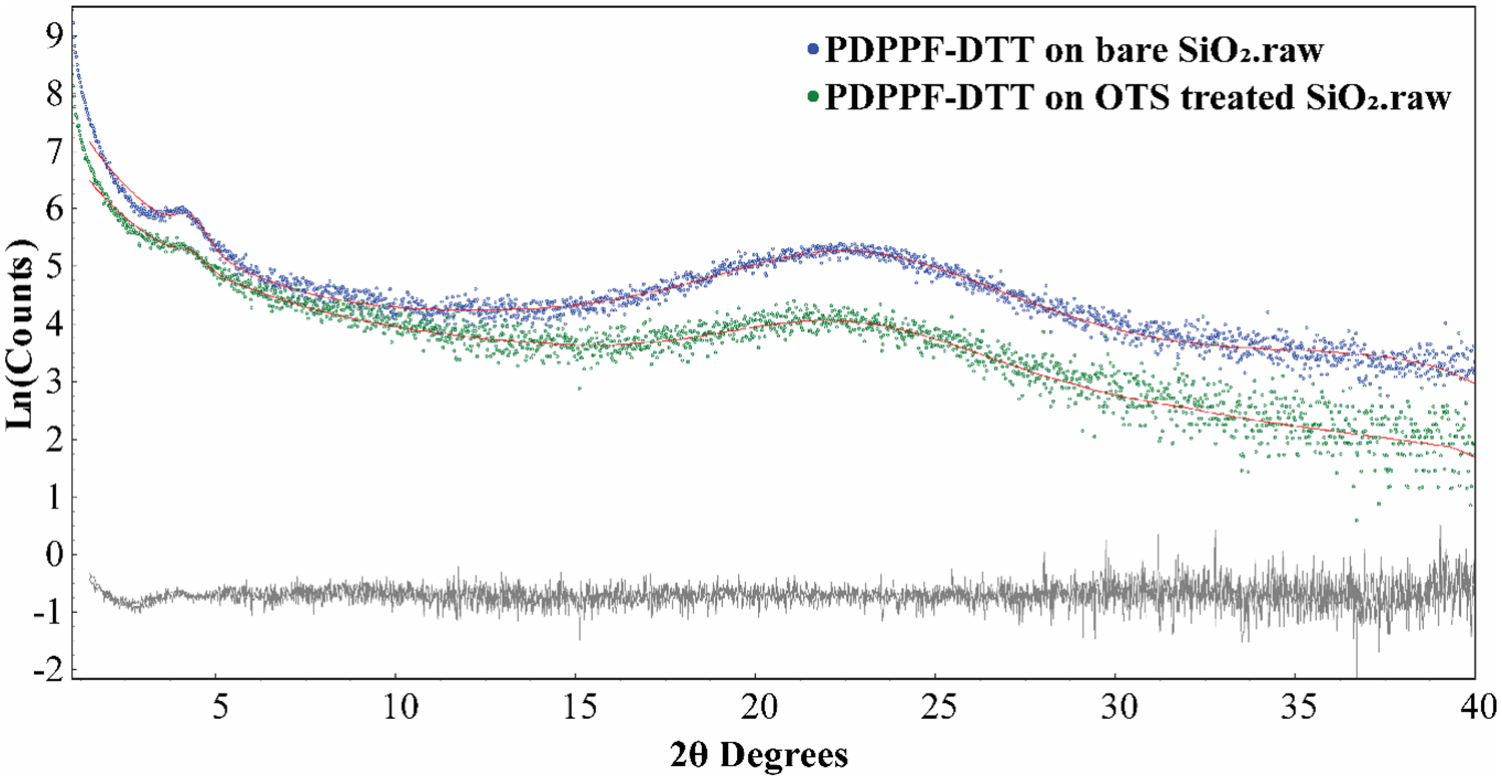
The fittings of two diffraction peaks in GID patterns of PDPPF-DTT thin film on untreated, and OTS treated SiO_2_ surfaces respectively, after annealing at 150 °C for 30 min. The intensities are shown in log scale.

After the systematic characterization of the bare and OTS treated SiO_2_ surfaces in terms of its hydrophobic nature and thin film morphology, we performed Grazing Incident Diffraction (GID) for PDPPF-DTT film grown on bear and OTS treated SiO_2_ surfaces, after annealed at 150 °C for 30 min to remove the solvent residues. Figure 3 shows the GID patterns of 2θ ranges between 1 to 40°. The relative broad diffraction peaks represent the nano-crystalline nature of the formed phase. Fig. S9 shows the diffraction peaks in GID on untreated Si/SiO_2_ substrate, without any polymer deposited is at around 53°. Thereby, the peaks at 4° and 22° are emerging from the PDPPF-DTT polymer. For the same polymer, the hump around 22° is discussed^38^. For further clarity the diffraction peaks around 4° and 22° on both patterns were fitted using Pseudo-Voigt^44^ type peaks phase in DIFFRAC.TOPAS v6 software^45^. The refined peak positions and full width half maxima (FWHM) are summarised in Table S1. The FWHM of the first peak between 3.1 to 5.5° 2θ are reduced from 0.81 to 0.70° 2θ upon OTS treatment; the FWHM of the second peak between 13 to 31° 2θ are reduced from 8.18 to 6.35° 2θ upon OTS treatment. Particularly in DPP class of polymers such a FWHM narrowing of the peaks generally implies that the lamellar structures have become considerably more uniform, which leads to a more uniform distribution of the d-spacing^46^. D-spacing can be calculated from peak positions following the Bragg’s law: d = λ/(2 Sinθ), where ‘d’ stands for the interplane spacing, ‘λ’ represents the radiation wavelength 1.54059 Å in our experiment, ‘θ’ represents the half of the peak position 2θ in the XRD measurement. Following OTS treatment, d-spacing values of these two peaks increased from 20.26 to 20.58 Å, and from 3.95 to 4.01 Å, respectively, indicating a larger unit cell. Hence, comparatively after OTS treatment, larger crystallite packing structure was formed, with increased nano-crystal size. This in turn leads to the enhancement of the increased mobility values from 9.49 × 10^−4^ to 3.77 × 10^−2^ cm^2^/Vs.

## OFET performance evaluation of PDPPF-DTT

**Figure 4:**
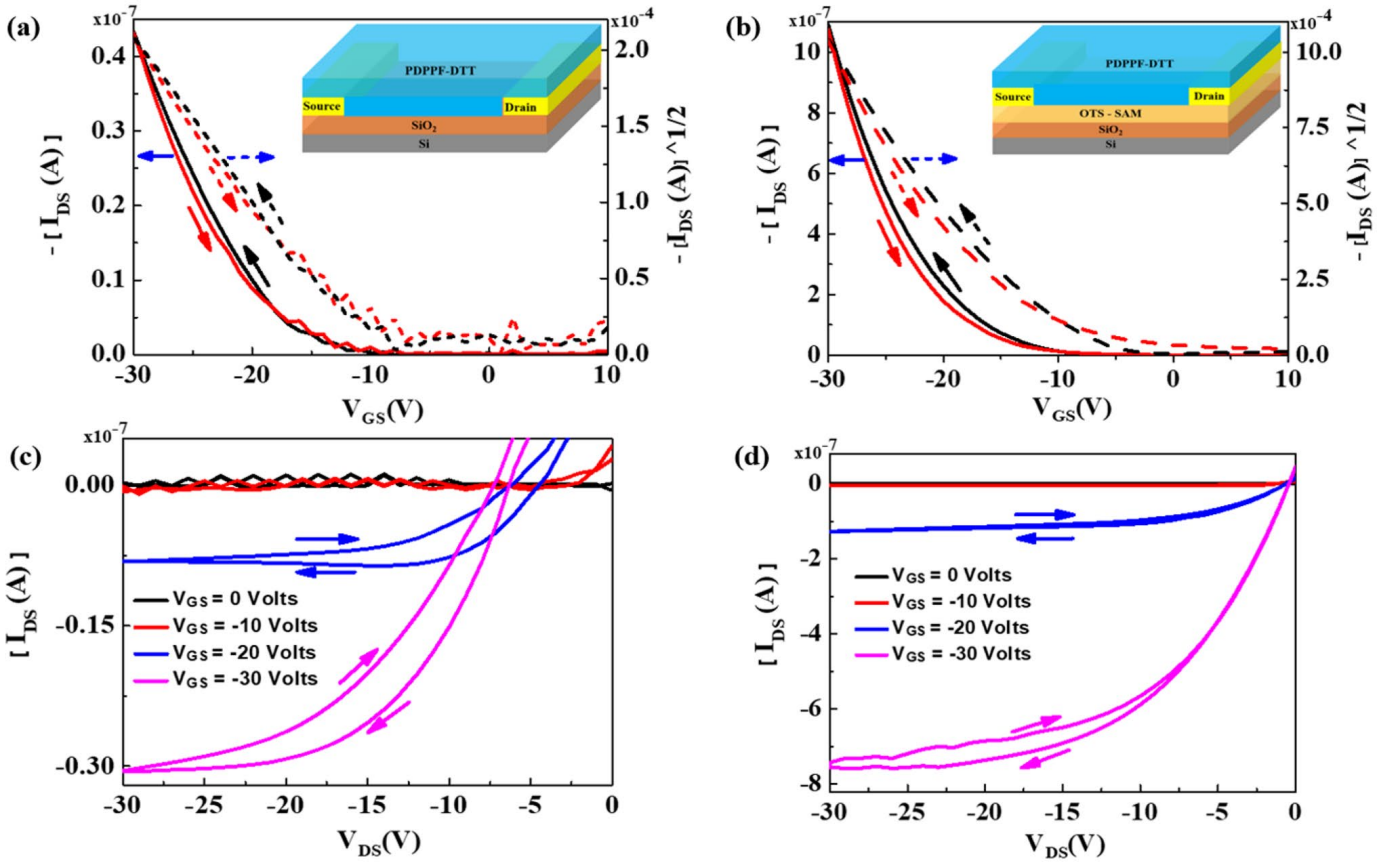
PDPPF-DTT based OFETs transfer (black line: forward; red line: reverse) and output characteristics as per (**a**,**c**) on Ref device type (without any surface treatment); and as per (**b**,**d**) on device type1 (with OTS surface treatment) respectively.

After thin film characterization of the PDPPF-DTT semiconducting film grown over bare and OTS treated SiO_2_ surface, we fabricated the complete OFET structure and the electrical characteristics taken out as output (I_DS_ − V_DS_) and transfer curves (I_DS_ − V_GS_). Figure 4a,c shows the transfer and output characteristics for OFETs fabricated bare SiO_2_ surfaces (Ref devices), while Fig. 4b,d shows the transfer and output characteristics for OFETs fabricated on OTS treated SiO_2_ surfaces (Type 1 devices). Log I_DS_ versus V_GS_ plots of PDPPF-DTT based OFETs on Ref and device type 1 are shown in Fig. S2(a) and Fig. S2(b), and that device of type 2 are shown in Fig. S3.

The basic equations explaining operation of OFETs in the linear, and in the saturation region is shown in the supplementary information, as per equation S1, and S2 respectively. The µ_FET_ can be extracted from the transfer characteristics of (I_DS_ vs. V_GS_), as in equation (S3) by plotting the square root of the saturation current as a function of V_GS_. Further the parameter SS narrates the important turn-on characteristics of the devices. This parameter is defined as the rate at which *I*_DS_ varies (in terms of decades) with *V*_GS_, while the device is operating at the subthreshold region. Thereby, it is extracted by fitting a line to the steepest portion of the subthreshold region, followed by taking inverse of obtained value, as narrated in equation (S4). All the device parameters for both cases are summarized in Table 1. This table showcases the significant enhancement in the mobility values of this polymer increases from 9.49 × 10^−4^ to 3.77 × 10^−2^ cm^2^/Vs, after OTS treatment. The obtained mobility values are slightly lower then PDPPT-DTT^4^, due to changes in the flanking group. The increased performance after the OTS Treatment can be attributed to enhanced crystallinity upon OTS treatment, confirmed by X-ray diffractogram and due increase in thin film surface roughness of the PDPPF-DTT from 3.68 nm on bare SiO_2_, to 4.42 nm after OTS treated SiO_2_.

**Table 1:** Summary of the obtained results of electrical performances parameters, for PDPPF-DTT OFETs. With, average values of the four devices.

Device type	(V_th_) (V)	(µ_FET_) (cm^2^/V s)	|I_on_/I _off_|	(SS) (V/dec)	${\varvec{N}}_{{{\varvec{Trap}}}}$ (/cm^2^)
Ref	− 15.42	8.83 × 10^−4^ (9.94 × 10^−4^)	3.86 × 10^2^	19.68	2.73 × 10^11^
1	− 15.80	3.43 × 10^−2^ (3.77 × 10^−2^)	1.58 × 10^3^	12.01	2.23 × 10^11^
2	5.74	8.70 × 10^−2^ (1.82 × 10^−1^)	4.03 × 10^1^	12.69	6.62 × 10^11^

Next, the V_th_ changes from − 15.42 to − 15.80 V after OTS treatment. The extent of change in V_th_ is larger than that observed in PDPPT-DTT^4^. Table 1. showcases the onset threshold voltage values and the average of three different OFET devices fabricated in a single substrate under similar conditions. To investigate the reasons, change in V_th_, we measured hysteresis in transfer characteristics for both, bare and OTS treated SiO_2_ surfaces. Hysteresis decrease after OTS treatment. This is measured by calculating the change in V_th_ in two cycles of the hysteresis. We calculated the trap density ($N_{Trap}$) in each case by using the following equation:^47^

1 $${\text{N}}_{{{\text{Trap}}}} = \frac{{{\text{C}}_{{\text{i}}} \left| {\Delta {\text{V}}_{{{\text{th}}}} } \right|}}{{\text{q}}}$$

wherein $C_{i}$ is dielectric capacitance and $q$ is an electronic charge. We obtained the trap density for bare and OTS treated OFET devices are 2.73 × 10^11^ cm^−2^ and 2.23 × 10^11^ cm^−2^, respectively. This reduced trap density confirms the good quality dielectric/semiconductor interface formation. But, on the other hand, the field due to trapped electron charges play an important role to control the net charge density in the OFET channel. Singh *et. al* studied the role of electron traps and their control over V_th_ in PVP/TIPS-pentacene based solution-processed OFETs. The field which arises due to electron traps at dielectric/semiconductor interface enhance the gate field, and hence leading to the reduction V_th_^47–49^. Field due to electron traps and their dynamics at dielectric–semiconductor interface is discussed by drain current transient measurements in^48,49^ and is schematically demonstrated in Fig. S5.

Further, we used the PFBT treatment of Au S/D electrodes over OTS treated SiO_2_ surfaces just before the deposition of PDPPF-DTT semiconductor film. We measured the work-function of Au by Photo-Electron Spectroscopy in Air (PESA). Riken AC-3 was used as the measuring system as showcased in Fig. S4. It was found the work function changes from 4.76 to 5.49 eV after PFBT treatment. Thereby, reducing the injection barrier between the electrode and semiconductor interface. In our case, the reduction in barrier height takes place from earlier ∆H_1_ = 0.59 eV to ∆H_2_ = 0.14 eV as show in Fig. 6a without PFBT treated Au, and in Fig. 6b with PFBT treated Au, respectively. This is consistent with previous OFET studies^24,27,50,51^.

**Figure 5:**
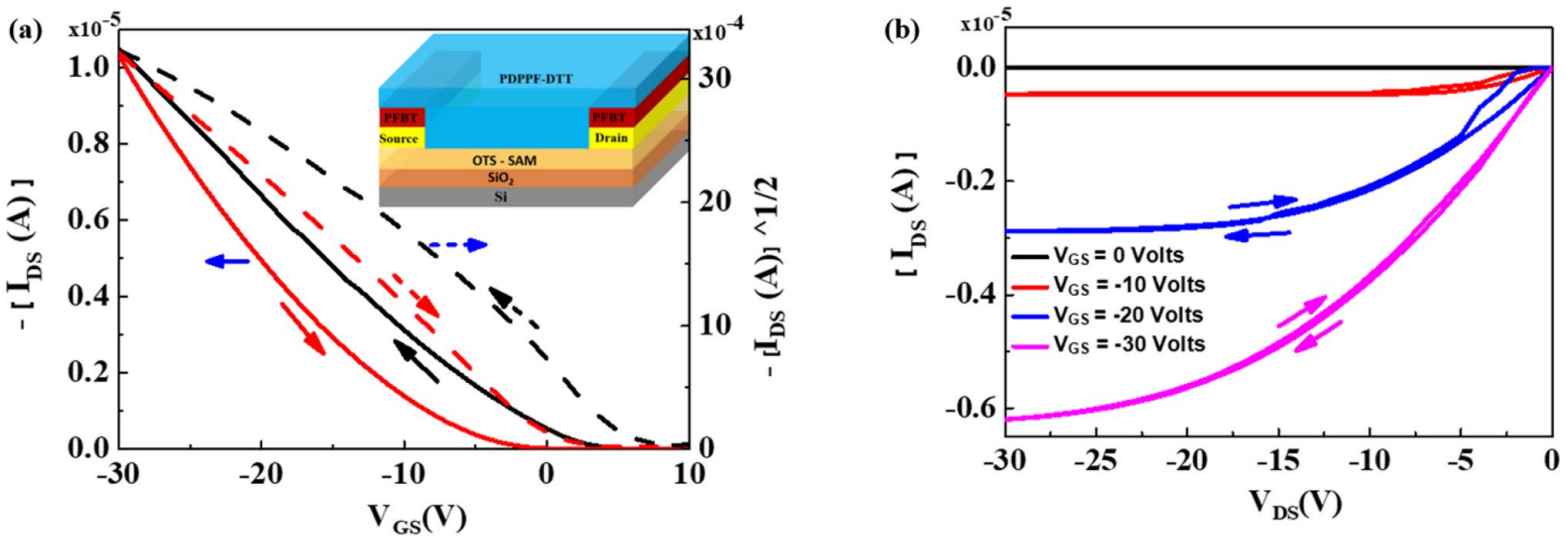
PDPPF-DTT based OFETs with PFBT on OTS treated surface (**a**) transfer (black line: forward; red line: reverse), and (**b**) output characteristics.

Figure 5a,b shows the transfer and output characteristics for OFETs fabricated on PFBT and OTS treated SiO_2_ surfaces (Type 2 devices). After PFBT treatment, mobility increases up to 0.182 cm^2^/Vs and V_th_ decreases to + 5.74 V. Both, mobility and V_th_ significantly change after PFBT treatment in comparison to bare and only OTS treated SiO_2_ surfaces. The obtained highest charge carrier mobility of 0.182 cm^2^/Vs for PDDPF-DTT upon OTS and PFBT dual treatment is slightly lower than earlier reported value and it could be attributed with different device geometry and interface effect. In our earlier report, we used top contact bottom gate devices but herein first time we used bottom gate bottom contact devices. Batch to batch variation in molecular weight and polydispersity of synthesized polymer may hamper on the performance and this is one of the major drawbacks of polymers compared to well defined small molecules or oligomers which has single molecular weight. In OFET devices, it has been reported that the charge carrier densities and the channel conductance could be strongly affected by the charge carrier injection barrier.

The tuning from negative V_th_ towards positive has been also observed in other types of organic semiconductor where PFBT was used as an interfacial layer with Au as source and drain electrode^27^. The significant increase in mobility in these type 2 devices (PFBT over OTS treated SiO_2_ surface) is expected to reduce the contact resistance after PFBT treatment. Few research groups^52,53^ reported the effect of contact resistance on charge carrier mobility for the bottom and top contact OFET geometry, lower contact resistance aids in to increased mobility.

**Figure 6:**
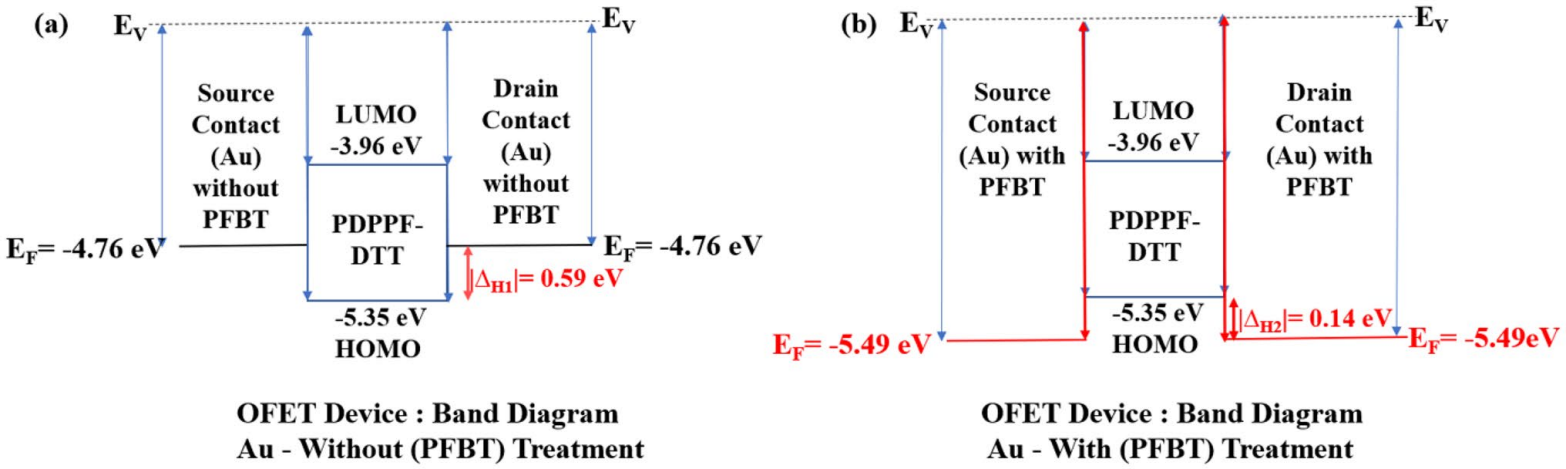
PDPPF-DTT based OFETs band diagram (**a**) without and (**b**) with PFBT treatment on Au surface.

Upon PFBT treatment average V_th_ reduces from − 15.42 to 5.74 V due reduced barrier height simultaneously increasing the mobility values. This reduction in V_th_ is significantly lower in comparison to bare and only OTS treated SiO_2_ surfaces. Our study points to significance of introducing traps in a selective manner that takes advantage of the presence of dipoles and the attendant electric field produced by them that can improve dissociation of both singlet and charge transfer excitons in organic devices^54–56^. Which is most recently debated by an in depth study by Abdu-Aguye et al.^57^. Thereby, we studied hysteresis in transfer characteristic for these type 2 devices as shown in Fig. 5a. The large counter—clockwise hysteresis has been observed. We calculated the trap density using Eq. (1). We achieve the trap density for these type 2 devices 6.62 × 10^11^ cm^−2^, which is very high in comparison to Ref. device, type 1 having values 2.73 × 10^11^ cm^−2^, and 2.23 × 10^11^ cm^−2^ respectively. The field occurred due to trapped interface electrons, aid in the process of enhancing the gate field, thereby causes charge accumulation at lower voltage (5.74 V in this case). This in turn thereby decreases I_on_/I_off_ ratio upon PFBT treatment (Fig. 6)^12^.

## Conclusions

In summary, following our work in^4^, we have optimised PDPPF-DTT polymer based p-type BGBC OFETs. SAM treatment was effectively done at dielectric layer– semiconductor and metal–semiconductor interfaces. Firstly, OTS treatment tuned the dielectric interface and enhanced crystallinity of the semiconducting thin film due to which highest obtained mobility increased by around two order of magnitude. Secondly, the injection barrier between Au S/D electrode and HOMO of the PDPPF-DTT polymer reduced by PFBT treatment, which further aided to enhance the highest obtained mobility of up to 0.18 cm^2^/Vs. At the same time, PFBT treatment increase the hysteresis in transfer characteristics, and estimated in terms of high electron trap density (6.62 × 10^11^ cm^−2^), which reduce V_th_ by controlling the field in channel from − 15.42 to 5.74 V. The positive shift in case of PDPPT-DTT^4^ was only up to 1 V, but in case of PDPPF-DTT it up to 5.74 V. Thereby, enhancing the performance further toward more positive value. Thereby we demonstrate a systematic possibility of reduction of the V_th_ with simultaneous enhancement in the mobility by effectively controlling the interface interaction phenomenon for the case of PDPPF-DTT.

## Experimental section

The BGBC devices were-fabricated on p^++^ highly doped silicon substrate with SiO_2_ as the dielectric layer with thickness of 200 nm, which was thoroughly cleaned by placing the substrates in Piranha solution, and heat at 80 °C for 10 min. Remove the Piranha solution and rinse the substrates with deionized (DI) water for three times. Sonicated with DI water, toluene, and isopropyl alcohol (IPA) for 10 min each in that order. Bake at 100 °C for 1 h. Exposure of the Si–SiO_2_ substrates to ethanol vapour at 150 °C. UV-Ozone (UV/O_3_) treatment for 10 min^58^. OTS treatment was performed by placing the cleaned Si–SiO_2_ substrates in OTS-chloroform 1:10 (500 µl/5 ml) solution for 6 h, after which the substrates were rinsed with the chloroform solvent, the obtained contact angle was (106° ± 2°). Bottom contact S/D Au metal of 70 nm-thick was deposited using the thermal vacuum deposition chamber. The channel length and channel width were 100 µm and 1.5 mm respectively, their deposited values was measured using laser microscope imaging, and the values obtained where used during measurement and further analysed^4^.

For devices requiring PFBT treatment, prior to spin coating of the organic semiconductor layer, the Au based source and drain electrode surfaces were modified accordingly. This PFBT Au modification was done by immersing the substrates in a 30 mM solution of PFBT in isopropanol for 5 min. Post which they were properly rinsed with pure isopropanol^59^. Au source–drain work function changes after PFBT treatment, was measured by Photo-Electron Spectroscopy in Air (PESA). For this measurement Riken AC-3 equipment was utilized.

During solution preparation PDPPF-DTT was used a solute in chloroform at ratio of 7 mg/ml and solution was prepared. During the same it was the magnetically stirred at 300 rpm for 20 min. Post which the formed solution was spin coated on the cleaned and appropriately treated substrates at 1000 rpm for 60 s, which was immediately followed by annealing them at 150 °C for 30 min. Then in the in the nitrogen environment, all the fabricated devices were characterized using Keithley 4200-SCS semiconductor parameter analyser. For thin film thickness measurement, the Dektak thickness profilometer was used. Bruker AFM analyser was the equipment used for thin film surface AFM analysis. Thin film XRD were measured in Rigaku SmartLab (CuKα Long Fine Focus Tube working at 40 kV 40 mA) in Grazing Incident Diffraction (GID) geometry. The 0.1 mm thick parallel beam grazing incident into thin film surface at 0.3°, taking surface information only. A 0.228° equatorial soller was used on the secondary side to allow only parallel beam go into a Hypix3000 detector working in 0D mode. 5° axial soller slit was used on the primary side. The data was scanned from 1° to 90° − 2θ, at 0.02° step size at scan speed 2° per minutes, by just scanning the detector in 2θ mode. Further, the electrical characteristics of the fabricated OFET were measured using a semiconductor parameter analyser in the N_2_ Glove Box Environment.

## Supplementary information

Supplementary Information 1.
