# Supplementary material for: Electrode and dielectric layer interface device engineering study using furan flanked diketopyrrolopyrrole–dithienothiophene polymer based organic transistors
Source: Sci Rep. 2020 Nov 17;10:19989. doi: 10.1038/s41598-020-76962-x (PMC7673034; doi:10.1038/s41598-020-76962-x)
Supplement: Supplementary file 1 — Supplementary Information 1. [file 41598_2020_76962_MOESM1_ESM.docx]

**Supplementary Information**

**Electrode and Dielectric Layer Interface Device Engineering Study Using Furan Flanked Diketopyrrolopyrrole-Dithienothiophene Polymer Based Organic Transistors**

Basanagouda. B. Patil,^a,c^ Yasunori Takeda,^c^ Subhash Singh,^c^ Tony.Wang,^e^ Amandeep Singh, ^b,e^ Thu Trang Do,^b^ Samarendra P. Singh,^d^ Shizuo Tokito,^c^* Ajay K. Pandey,^a^* Prashant Sonar,^b,e^ *

^a^ School of Electrical Engineering and Robotics, Science and Engineering Faculty, Queensland University of Technology (QUT), Brisbane, QLD 4000, Australia

^b^ School of Chemistry, Physics and Mechanical Engineering, Science and Engineering Faculty, Queensland University of Technology (QUT), Brisbane, QLD 4000, Australia.

^c^ Research Center for Organic Electronics (ROEL), Yamagata University, 4-3-16 Jonan, Yonezawa, Yamagata, 992- 8510, Japan

^d^ Department of Physics, School of Natural Sciences, Shiv Nadar University (SNU), Gautam Buddha Nagar, Uttar Pradesh, India-201307

^e.^ Centre for Material Science, Queensland University of Technology, Brisbane, QLD 4000, Australia

Corresponding Author Emails : [sonar.prashant@qut.edu.au](mailto:sonar.prashant@qut.edu.au), [a2.pandey@qut.edu.au](mailto:a2.pandey@qut.edu.au), [samarendra.singh@snu.edu.in](mailto:samarendra.singh@snu.edu.in), [tokito@yz.yamagata-u.ac.jp](mailto:tokito@yz.yamagata-u.ac.jp)


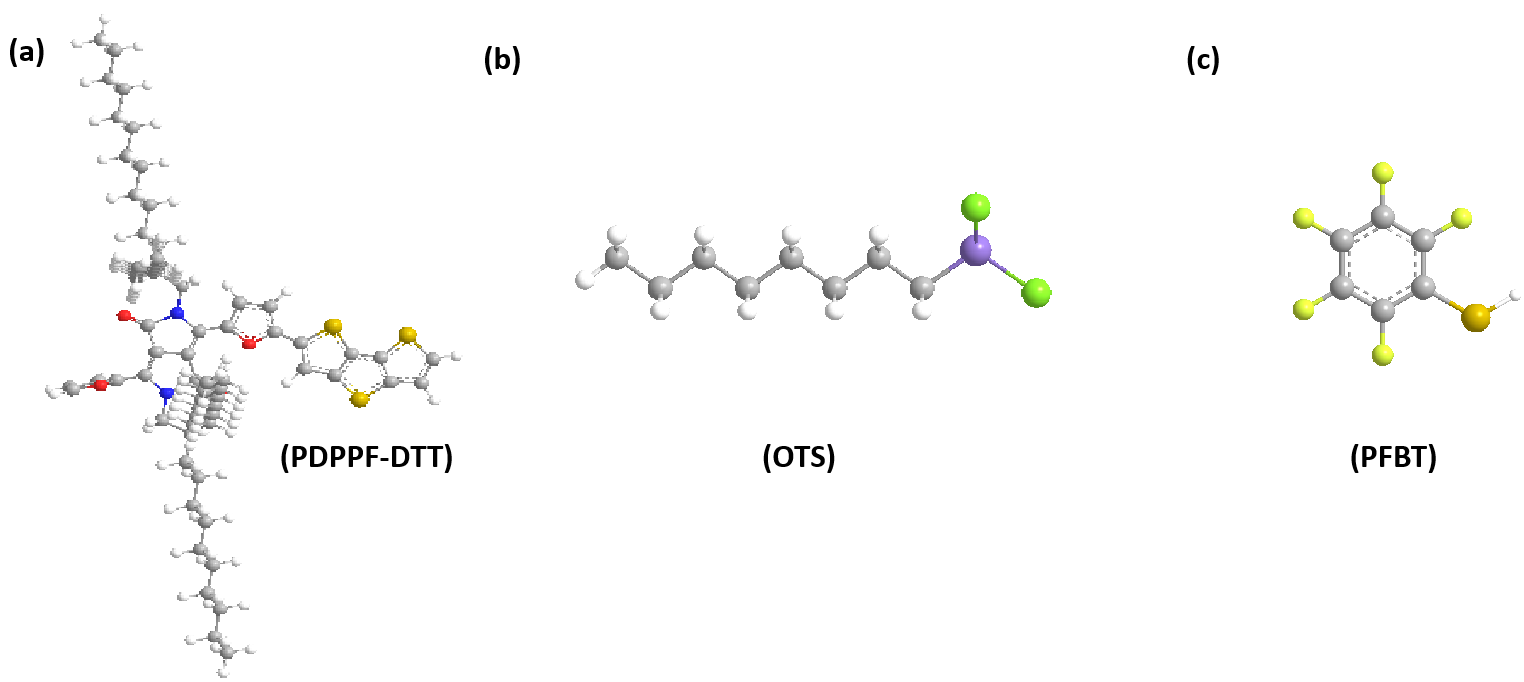


**Figure S1:** 3-D molecular structures of (a) PDPPF-DTT**,** (b) (OTS) and (c) PFBT


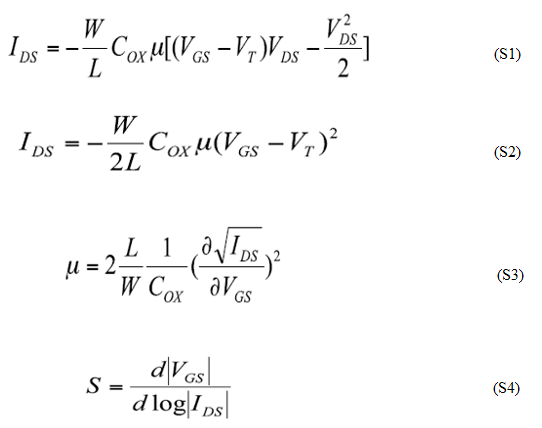


**Table S1** Grazing Incident Diffraction (GID) analysis of PDPPF-DTT on Untreated, and OTS treated surfaces respectively after annealing at 150°C for 30 minutes

| ­­Thin Film XRD | Peak range from 3.1 ~ 5.5° 2θ° | | | | Peak range from 13 ~ 31° 2θ° | | | |
| --- | --- | --- | --- | --- | --- | --- | --- | --- |
| Surface and Polymer Type | **Peak Intensity (Counts)** | **Peak position (2θ**°**)** | **FWHM (2θ**°**)** | **D -Spacing (Å)** | **Peak Intensity (Counts)** | **Peak position (2θ**°**)** | **FWHM (2θ**°**)** | **D –Spacing (Å)** |
| PDPPF-DTT  On Untreated SiO_2_ Surface | 137.33 | 4.36 | 0.81 | 20.26 | 162.50 | 22.52 | 8.18 | 3.95 |
| PDPPF-DTT  On OTS SiO_2_ Surface | 61.72 | 4.23 | 0.70 | 20.97 | 59.71 | 22.15 | 6.35 | 4.01 |


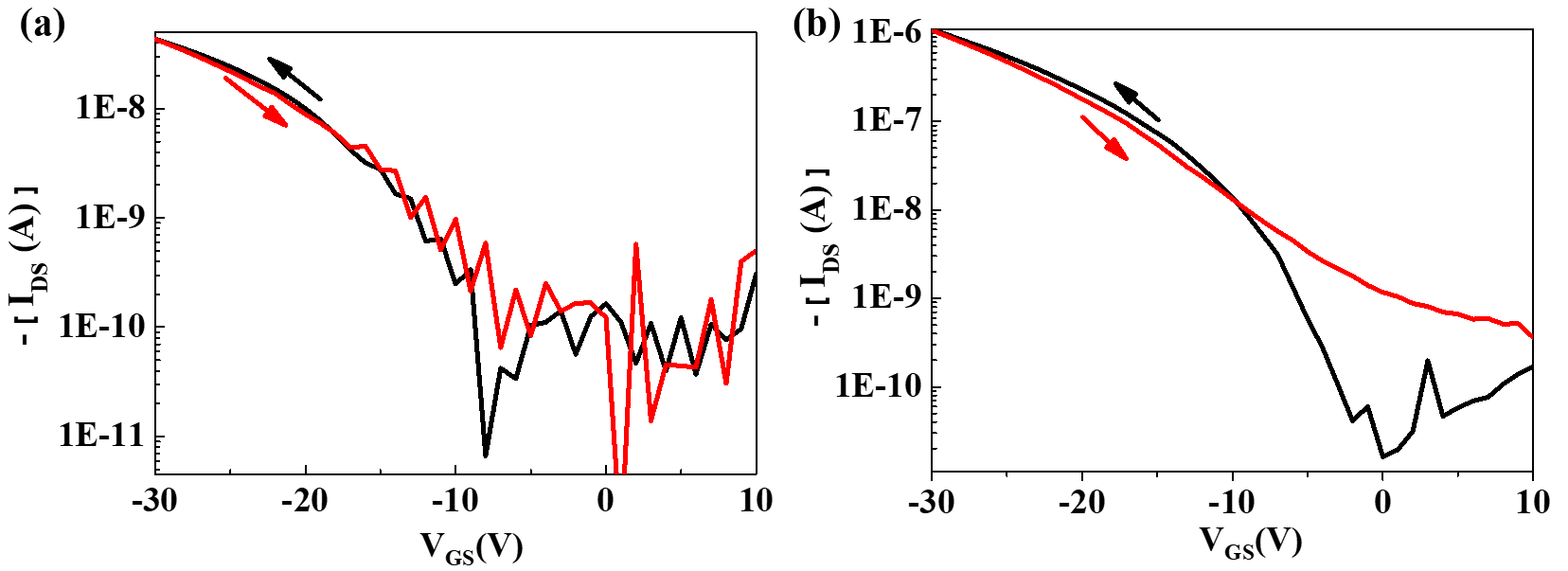


**Figure S2:** Log I_DS_ vs V_GS_ plots of PDPPF-DTT based OFETs transfer (black line: forward; red line: reverse). Wherein, (a) is on Ref device type (without any surface treatment); and (b) is on device type1 (with OTS surface treatment).


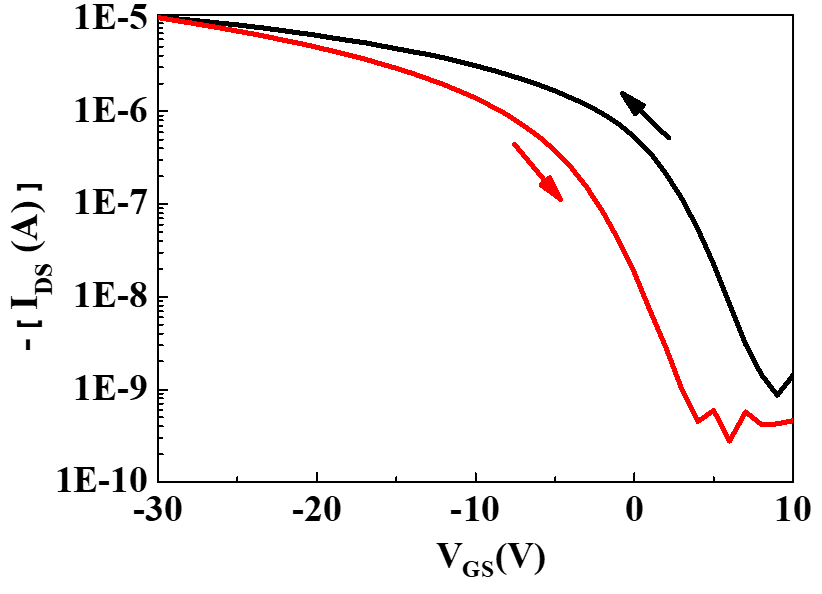


**Figure S3:** Log I_DS_ vs V_GS_ plots PDPPF-DTT based OFETs on device type 3, with both PFBT on OTS Treated surface transfer (black line: forward; red line: reverse).


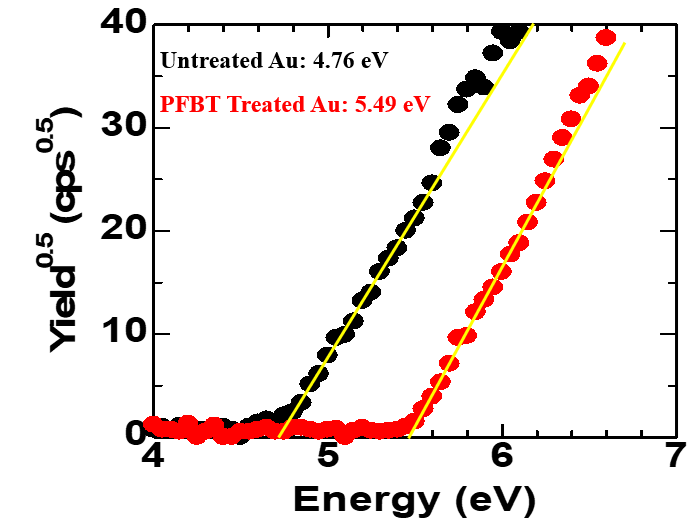


**Figure S4:** Au Source-Drain Metal Work function changes measured by Photo-Electron Spectroscopy in Air (PESA) with help of Riken AC-3 equipment

**Figure S5**. Schematic diagram of the net field contribution from gate field (larger arrow), and field due to traps (smaller arrow). E_C_, E_T_, and E_V_, are conduction band, trap energy level, and valence band, respectively.


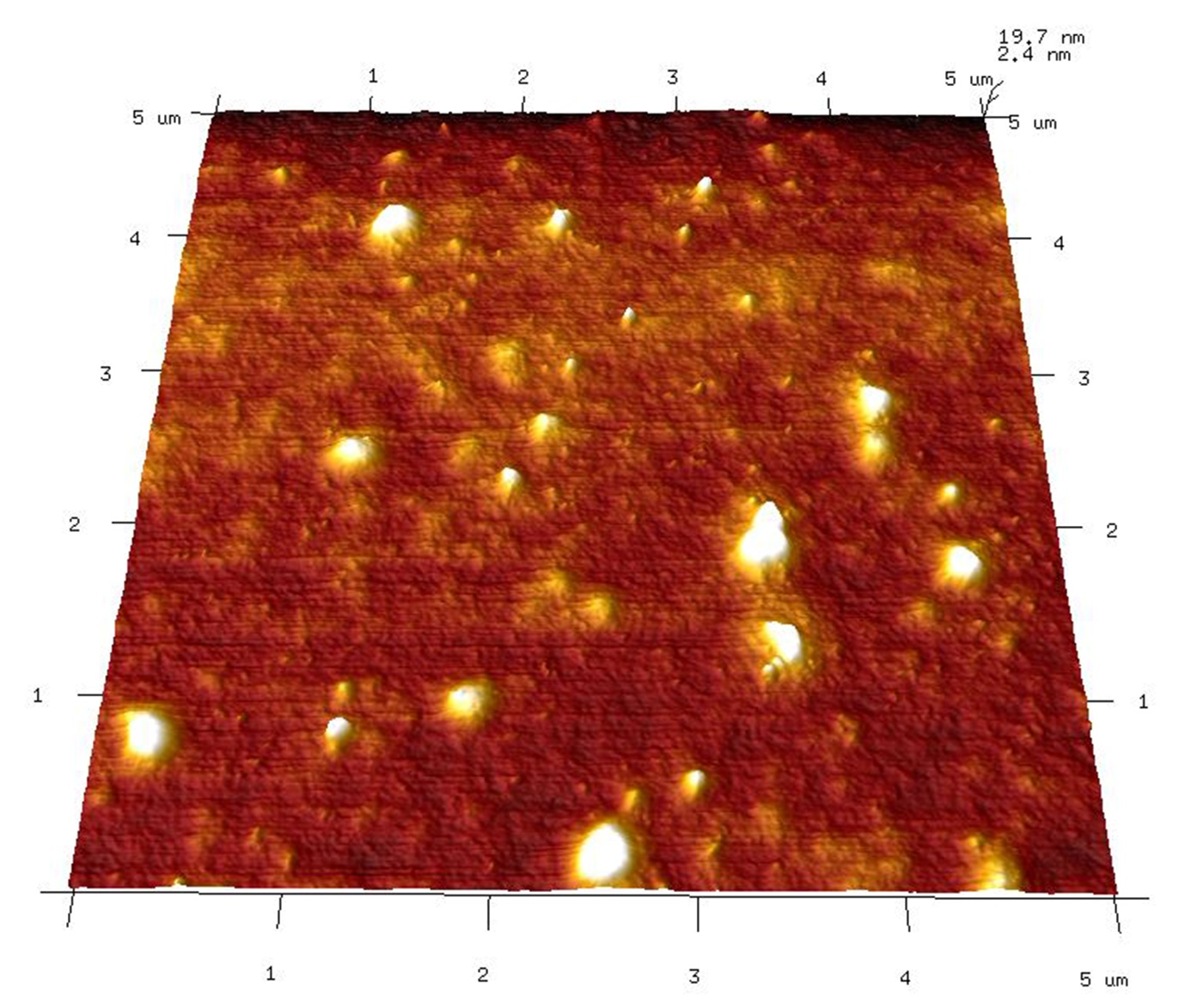


**Figure S6**. Large Size AFM images (5µm × 5µm surface area, and scale is +10nm to -10nm) of PDPPF-DTT, after annealing at 150ºC for 30 minutes with average nominal height of 3.68 nm for untreated, SiO_2_ surfaces


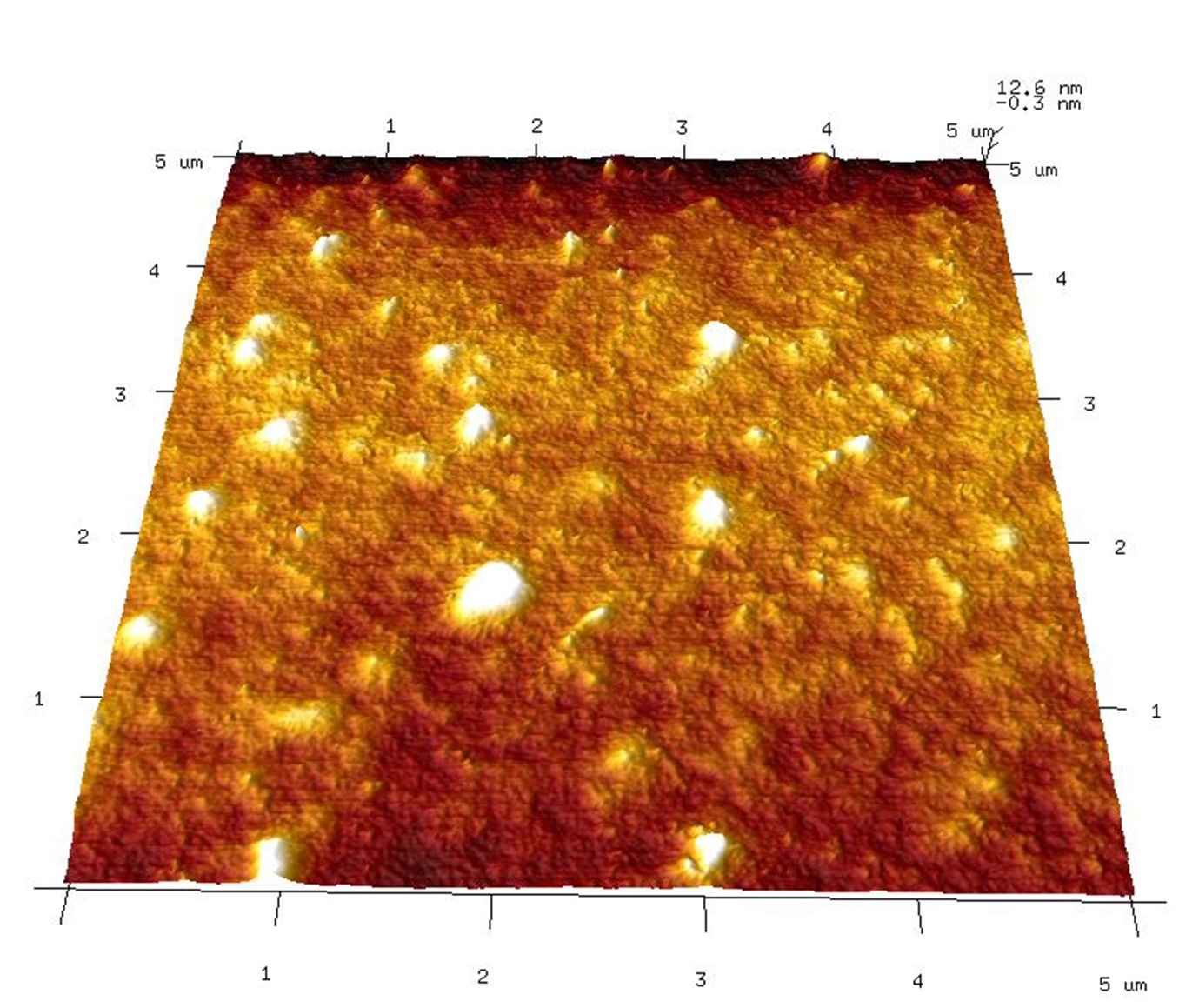


**Figure S7**. Larger size AFM images (5µm × 5µm surface area, and scale is +10nm to -10nm) of PDPPF-DTT, after annealing at 150ºC for 30 minutes with average nominal height of 4.42 nm for OTS treated SiO_2_ surfaces.


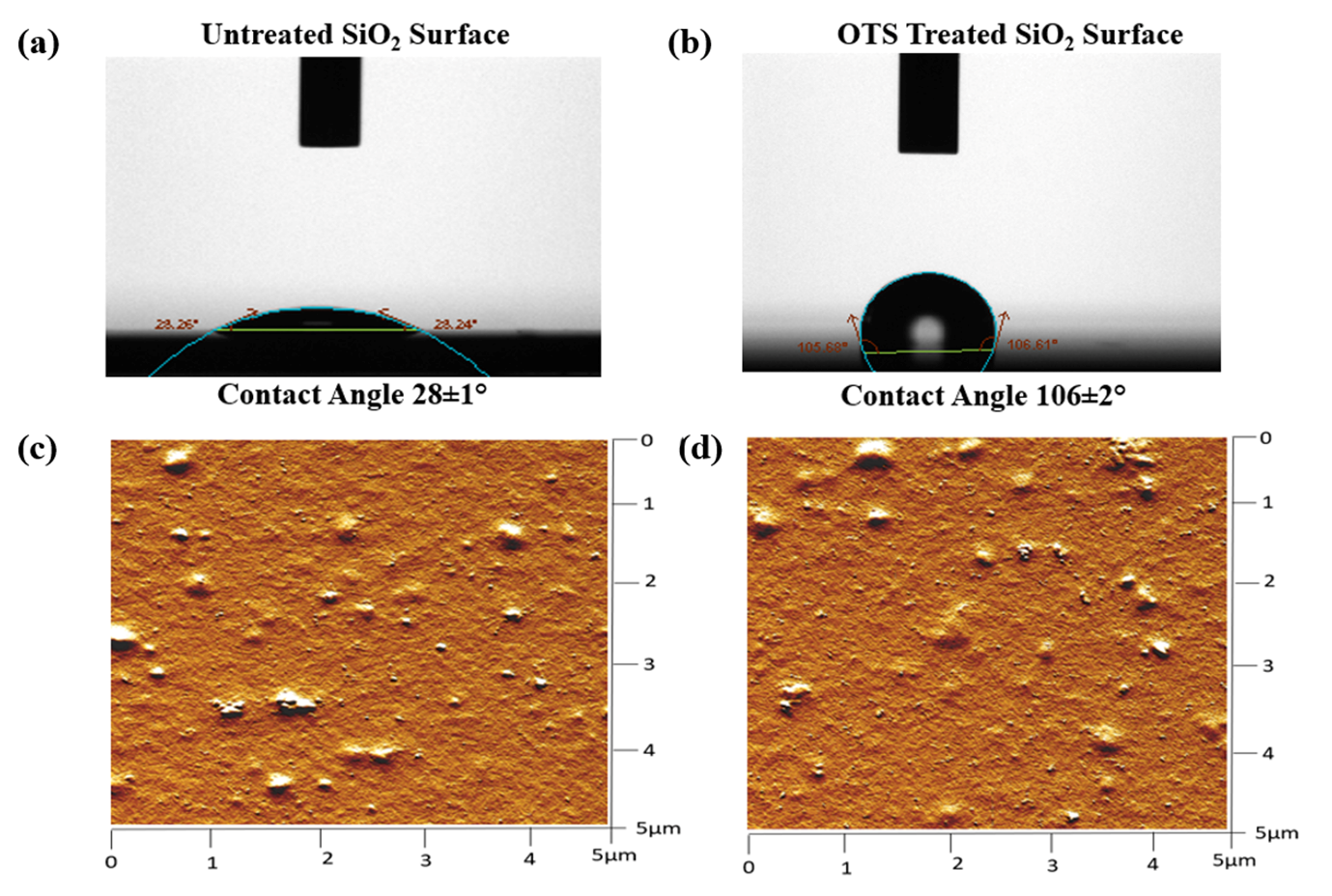


**Figure S8**. SiO_2_ surface contact angle of (a) 28±1°, for untreated, and (b) 106±2°, for OTS treated SiO_2_ surfaces respectively. AFM images (5µm × 5µm surface area, and scale is +10nm to -10nm) of PDPPF-DTT, after annealing at 150ºC for 30 minutes with average nominal height of (c) 3.68 nm for untreated, and (d) 4.42 nm for OTS treated SiO_2_ surfaces, respectively.


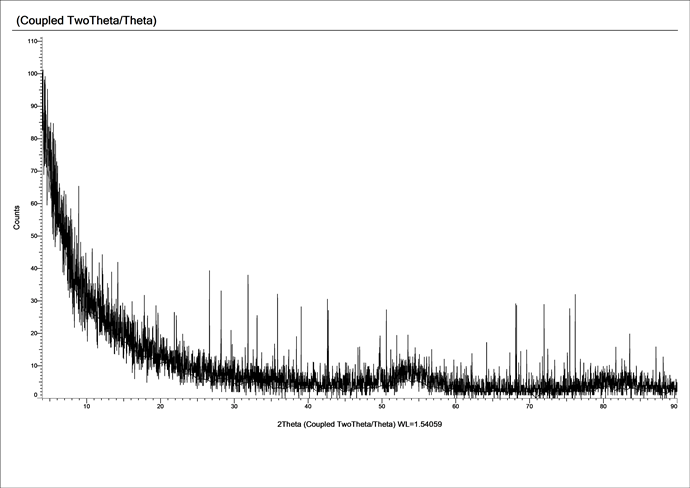


**Figure S9:** The diffraction peaks in Grazing Incident Diffraction (GID) on untreated Si/SiO2 substrate, without any polymer deposited. Thereby showcasing the 2theta diffraction hump in case of SiO_2_, is around 53^o^ and no where else.
